# The Influence of Preprocessing Steps on Graph Theory Measures Derived from Resting State fMRI

**DOI:** 10.3389/fncom.2018.00008

**Published:** 2018-02-13

**Authors:** Fatma Gargouri, Fathi Kallel, Sebastien Delphine, Ahmed Ben Hamida, Stéphane Lehéricy, Romain Valabregue

**Affiliations:** ^1^Institut du Cerveau et de la Moelle Épinière, Centre de NeuroImagerie de Recherche, Paris, France; ^2^Sorbonne Universités, UPMC Univ Paris 06, Institut National de la Santé et de la Recherche Médicale, Centre National de la Recherche Scientifique UMR 7225, Paris, France; ^3^Advanced Technologies for Medicine and Signals, ENIS, Université de Sfax, Sfax, Tunisia

**Keywords:** graph theory, preprocessing, resting-state fMRI, control quality, tCompCor

## Abstract

Resting state functional MRI (rs-fMRI) is an imaging technique that allows the spontaneous activity of the brain to be measured. Measures of functional connectivity highly depend on the quality of the BOLD signal data processing. In this study, our aim was to study the influence of preprocessing steps and their order of application on small-world topology and their efficiency in resting state fMRI data analysis using graph theory. We applied the most standard preprocessing steps: slice-timing, realign, smoothing, filtering, and the tCompCor method. In particular, we were interested in how preprocessing can retain the small-world economic properties and how to maximize the local and global efficiency of a network while minimizing the cost. Tests that we conducted in 54 healthy subjects showed that the choice and ordering of preprocessing steps impacted the graph measures. We found that the **csr** (where we applied realignment, smoothing, and tCompCor as a final step) and the **scr** (where we applied realignment, tCompCor and smoothing as a final step) strategies had the highest mean values of global efficiency ***(eg)***. Furthermore, we found that the **fscr** strategy (where we applied realignment, tCompCor, smoothing, and filtering as a final step), had the highest mean local efficiency ***(el)*** values. These results confirm that the graph theory measures of functional connectivity depend on the ordering of the processing steps, with the best results being obtained using smoothing and tCompCor as the final steps for global efficiency with additional filtering for local efficiency.

## Introduction

Resting state functional MRI (rs-fMRI) is an imaging technique that allows measuring the spontaneous fluctuations of the blood oxygen level-dependent (BOLD) signal in the brain. This technique has revealed the permanent existence of several networks in healthy subjects that are identifiable through their functional connectivity (Biswal et al., 1995). Functional connectivity is defined by the synchronization of the BOLD signal changes between distant regions. Measures of connectivity have been used as biomarkers to identify the various normal and pathological behavioral or cognitive states of the brain (Bassett et al., 2009; Bullmore and Sporns, 2009, 2012). Cerebral networks have small-world characteristics with highly clustered local connectivity and relatively few long-distance connections (Watts and Strogatz, 1998; Bassett and Bullmore, 2006). The economical properties of small-world networks are characterized by having high local and global efficiency at a low cost (Latora and Marchiori, 2001, 2003). These properties have been studied using graph theory, which is increasingly used to evaluate healthy and pathological brains (Bassett et al., 2009; Bullmore and Sporns, 2009, 2012) and has shown acceptable reliability (Wang et al., 2010; Guo et al., 2012; Telesford et al., 2013).

BOLD signal changes at rest are also sensitive to artifacts. Therefore, measures of functional connectivity are highly dependent on the quality of the BOLD signal data processing. Several studies have investigated the reliability and reproducibility of rs-fMRI and reported the importance of measuring this reliability (Bennett and Miller, 2010). They also noted that the reliability of fMRI data was low compared to other imaging measures and needed much more processing steps to improve reliability estimates. The reliability of rs-fMRI has been studied using several measures of functional connectivity (Zuo and Xing, 2014). Studies emphasized the need to ensure low variability among the subjects in the same group and high variability between the subjects of different groups (Zuo and Xing, 2014). They also discussed that the choice in the different preprocessing strategies can affect the reliability of rs-fMRI. Moreover, they showed that the final results also depended on the post-preprocessing methods (seed-based analysis, independent component analysis, graph theory, etc.).

Several approaches have been proposed to improve the quality of rs-fMRI images and more specifically to correct for artifacts. The frequency filtering of data using a Butterworth filter with a bandwidth of 0.01–0.1 Hz eliminated high and low frequencies (Biswal et al., 1995; Garreffa et al., 2003). Regression of the global cerebral signal can also be applied to isolate the relevant neural signal (Vincent et al., 2006; Bettus et al., 2009) and regression of the signal of the white matter and the cerebrospinal fluid can be used as a complementary technique to minimize the influence of artifacts on correlation maps (Bartels and Zeki, 2005). In addition, there are methods for head movement artifact removal during acquisition, besides realignment (Van Dijk et al., 2012; Satterthwaite et al., 2013; Power et al., 2014). The measure “Framewise Displacement” (FD) was proposed to identify the volumes where subjects show high head movements (Power et al., 2014). Another component-based method (CompCor) has been reported that reduced two artifacts (physiological noise and head movement) in the functional data (Behzadi et al., 2007).

However, as mentioned above, the way preprocessing is applied may influence the final results. For instance, the use of global signal regression may increase the correlation between the measures of connectivity and head movement (Jo et al., 2013). Other authors confirmed that the use of global signal regression decreased the reliability of rs-fMRI using graph theory measures (Liang et al., 2012) and matrix correlation (Guo et al., 2012). Another study evaluated the reliability of graph theory measures and showed that the use of global signal regression increased the number of negative correlations (Braun et al., 2012), introduced spurious anticorrelations (Murphy et al., 2009) and caused an overestimation of functional connectivity strengths (Weissenbacher et al., 2009). The test-retest reliability of fMRI using graph theory measures were high when slow-4 filtering was applied (Liang et al., 2012) and when this step was applied with no detrending and no global signal regression (Borchardt et al., 2016). Head movements may introduce false positives when estimating functional connectivity (Van Dijk et al., 2012; Satterthwaite et al., 2013; Power et al., 2014). The evaluation of the relationship between measures of graph theory and head motion showed that this dependence decreased at the group level, as opposed to the individual level where there was no improvement (Yan et al., 2013b). Other authors evaluated the reliability of different preprocessing methods to estimate measures of graph theory and found that the use of scrubbing reduced the dependence between graph theory measures and head motion (Aurich et al., 2015).

The choice of preprocessing steps is very important. Choosing the most appropriate one is difficult. The preprocessing steps influenced the final functional connectivity through either a seed-based analysis (Chang and Glover, 2009; Weissenbacher et al., 2009) or graph theory (Yan et al., 2013a,b; Aurich et al., 2015). Additionally, it has been shown that the topological network differences between healthy volunteers and patients were highly dependent on the preprocessing steps (Borchardt et al., 2016). Therefore, more work has been done to evaluate the influence of global regression signal and filtering on functional connectivity.

In our project, we aimed to study the influence of classical preprocessing steps, including slice-timing, realignment, smoothing, and filtering in addition to tCompCor (Behzadi et al., 2007) on graph theory measures. We were particularly interested in studying the influence of the application order of these preprocessing steps on graph theory measures, particularly the global and local efficiency.

## Methods

### Defining strategies

**Figure 1:**
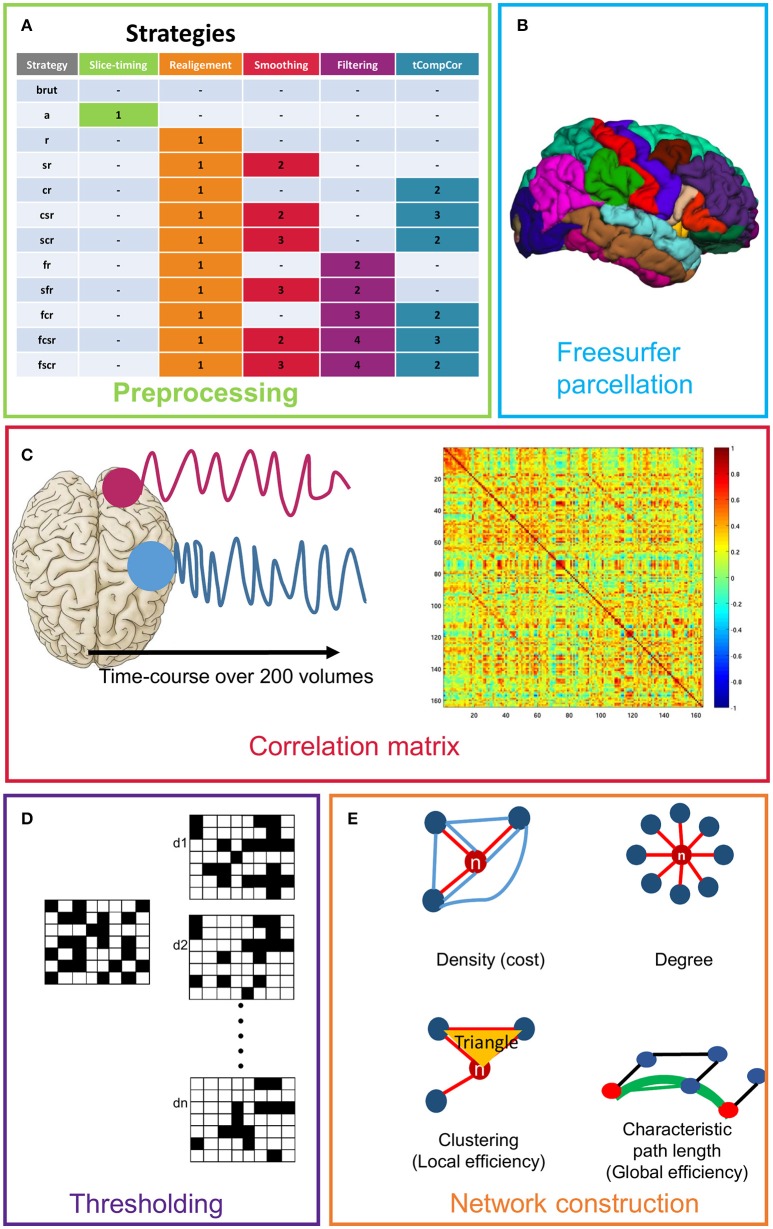
Presentation of the different steps used for network creation and definition of the different strategies. **(A)** Presentation of all strategies (we defined the order of application of each step). **(B)** Freesurfer parcellation (only the cortical regions are shown). **(C)** Time course calculation and example of a correlation matrix. **(D)** Simulation of the thresholding of the correlation matrix over a range of density (d1,.dn). **(E)** Representative diagram of the used graph theory measures.

Functional data were preprocessed using different steps. The analysis was performed in the native space. Realignment of the structural T1-weighted volume on the functional reference scan was performed using SPM8 in the native functional space. We defined seven different strategies for functional preprocessing, which are detailed in Figure 1.

Strategy **brut**

In this strategy, we considered the data without any preprocessing. We also defined a *strategy **a***, in which we applied only slice-timing. In view of the results that did not show any significant influence of slice-timing, we chose the following strategies that did not include slice-timing correction.

Strategy **r**

We only applied realignment to correct head movements, which occurred during the acquisition of the volumes to evaluate the impact of this step on the final result. A first volume in the functional series was taken as a reference. Each image of the time series was therefore processed according to a rigid displacement toward the chosen reference image.

Strategy **sr**

We applied realignment and then performed a smoothing using a 5-mm width at half maximum Gaussian filter.

Strategy **cr**

We applied realignment and then the component based noise correction method (tCompCor), which allowed the reduction of the physiological noise and head motion in the functional data (Behzadi et al., 2007). In this algorithm, the voxels dominated by physiological noise were processed using the temporal standard deviation (tSTD) of the time courses. A matrix including calculated times courses was processed using the principal component analysis (PCA) algorithm for component identification and classification. From this, we defined three new strategies.

Strategy **csr**

We applied realignment, smoothing, and then tCompCor.

Strategy **scr**

We applied realignment, then tCompCor and finally smoothing.

Strategy **fr**

We applied **strategy r** and then filtering (0.01 Hz, 0.1 Hz).

Strategy **sfr**

We applied **strategy fr** and then smoothing.

Strategy **fcr**

We applied **strategy cr** and filtering as the final step (0.01 Hz, 0.1 Hz).

Strategy **fcsr**

We applied **strategy csr** and filtering as the final step (0.01 Hz, 0.1 Hz).

Strategy **fscr**

We applied **strategy scr** and filtering as the final step (0.01 Hz, 0.1 Hz).

### Subjects & image acquisition

We considered 54 healthy volunteers (HV) in this fMRI analysis. All HV had no history of any neurological or psychiatric disease and did not present any contraindications for MRI. For data acquisition, we used a 3T Siemens Trio with body coil excitation and a 12-channel head coil for signal reception. Acquisition of anatomical images was performed using a sagittal three-dimensional T1-weighted Magnetization-Prepared Rapid Acquisition Gradient Echo (MPRAGE) acquisition characterized by a field of view (FOV) = 256 × 256 mm^2^, TR = 2,200 ms, echo time (TE) = 2.9 ms, flip angle = 10°, and a voxel size = 1 × 1 × 1 mm^3^.

The acquisition of rs-fMRI data of the whole brain was performed using a gradient echo echo-planar imaging sequence sensitive to BOLD signal with the following parameter: matrix size = 64 × 64, 45 slices, TR = 2400 ms, TE = 30 ms; flip angle = 90°, 200 volumes in one session, voxel size = 3 × 3 × 3 mm^3^ without gap, acquisition time = 8 min. Subjects were instructed to relax and completely close their eyes without sleeping during the scanning sessions.

We calculated the FD for each subject (Power et al., 2012) and we excluded each subject having a FD > 0.05 mm for at least one volume. Four subjects were thus excluded from the analysis. The HV were included at the (Institut du Cerveau et la Moelle epinière, Paris, France). The study was approved by the local ethics committee, and all of the participants provided written, informed consent prior to participating in the study.

### Network construction

For the construction of the brain networks, we defined 164 regions of interest (ROIs) as a first step, according to an anatomical model (Alexander et al., 1986), all of which were obtained through parcellation using FreeSurfer (Fischl et al., 1999, 2004) and covering the main cortical and subcortical areas. For each subject, we applied a cortical reconstruction using the spherical transformation of FreeSurfer. The functional network (or graph) was represented as nodes interconnected by links. It was constructed for each subject as follows: the nodes represented the ROIs and the links were the correlation between the mean signals of all the pairs of nodes (Figure 1).

To assess the small-world properties in the networks, we computed the small-worldness parameters for each graph (Humphries and Gurney, 2008; Rubinov and Sporns, 2010). These measures were designed for unweighted graphs and were highly dependent on the graph cost, which corresponded to the graph's density. For analyzing the topological properties of the brain functional networks, it was necessary to calculate the binary graphs which were obtained by thresholding each obtained correlation matrix. A common network costs should be able to mathematically compare the topological measures across all considered subjects. Successive thresholds of functional connectivity matrices were established over a range of network costs (Achard and Bullmore, 2007). We calculated the average values of the obtained topological results of the various metrics estimated for each individual network over the available cost range 0.04 and 0.25.

We estimated the small-worldness σ of the whole brain network at each cost for each strategy. We defined the upper limit of the small world regime as the highest cost (*K* = 0.25) at which the minimum value of σ was >1 for all the strategies (Bassett et al., 2008; Messé et al., 2013).

### Quantification of graph theory measures

Graph theory measures were calculated for each subject and for each strategy. To be able to compare the topologies of the networks for each strategy, we also calculated the graph theory measures for the two extreme networks: regular and random. The Brain Connectivity Toolbox was used to compute the graph theory measures (http://www.brain-connectivity-toolbox.net) (Rubinov and Sporns, 2010).

First, we calculated the small-worldness coefficient “σ” using equation 1 to check if the considered networks had small-world properties. A network was considered to have small-word properties if σ > 1 (Humphries and Gurney, 2008). These authors demonstrated that in the real world, there was no maximization of the value of σ. Therefore, the best strategy did not necessarily provide the greatest value of “σ” but rather the value that maintained its model more for the density interval between 0.04 and 0.25.

(1) $$\begin{array}{l} \sigma =\frac{\frac{C}{C_{rand}}}{\frac{L}{L_{rand}}} \end{array}$$

where *C, C*_*rand*_, *L*, and *L*_*rand*_ represented the clustering coefficients and the characteristic path lengths of tested and random networks, respectively.

The density or cost was the actual number of edges in the graph as a proportion of the total number of possible edges (Bullmore and Sporns, 2009).

(2) $$\begin{array}{l} K=\frac{l}{\left(N^{2}-N\right)/2} \end{array}$$

where *l* was the number of links in the graph, *N* was the number of nodes, and *N (N-1)/2* was the maximum number of links.

The characteristic path length of the network “*L*” corresponded to the average distance between the nodes *i* and all other nodes and was calculated using Equation (4).

(3) $$\begin{array}{l} L=\frac{1}{n}\sum L_{i}=\frac{1}{n}\sum \left(\frac{\sum d_{ij}}{n-1}\right) \end{array}$$

where *N* represented the set of all nodes in the network, *n* was the number of nodes and *d*_*ij*_ was the shortest path between the *i* and *j* nodes.

The clustering coefficient “C” corresponded to the fraction of a node's neighbors that were also neighbors of one other and was calculated using Equation (5).

(4) $$\begin{array}{l} C=\frac{1}{n}\sum C_{i}=\frac{1}{n}\sum \left(\frac{2t_{i}}{k_{i}\left(k_{i}-1\right)}\right) \end{array}$$

where *k*_*i*_ and *t*_*i*_ represented the degree of a node and the number of connections for a given node, respectively.

The local efficiency and clustering coefficient were used to evaluate network ability for processing specialized information within densely interconnected groups of nodes (functional segregation). The higher the clustering coefficient and local efficiency, the more segregated the network.

The local efficiency “*el*” reflected the local information transfer among the nodes and represented the robustness of the node to the deletion of individual nodes (Latora and Marchiori, 2001). ***el*** was calculated according to Equation (6).

(5) $$el=\frac{1}{n}\sum el_{i}=\frac{1}{n}\sum \frac{\sum a_{ij}a_{ih}{\left[d_{jh}\left(N_{i}\right)\right]}^{-1}}{k_{i}-\left(k_{i}-1\right)}$$

where *el*_*i*_ was the local efficiency of the node *i*, and *d*_*jh*_*(N*_*i*_*)* was the length of the shortest path between *j* and *h* and contained only the neighbors of *i*.

The global efficiency “*eg*” of the network was calculated according to Equation (7) (Latora and Marchiori, 2001).

(6) $$\begin{array}{l} eg=\frac{1}{n}\sum eg_{i}=\frac{1}{n}\sum \frac{\sum d_{ij}^{-1}}{n-1} \end{array}$$

where *eg*_*i*_ was the efficiency of node *i*.

As shown by Achard and Bullmore (2007), small-world networks may also be defined as having high global and local efficiency of parallel information transfer. In our project, we evaluated how the values of ***el*** and ***eg*** depended on the preprocessing strategies. We assumed that the best strategy would provide the highest values of these two measures.

### Statistical analysis

We compared graph theory measures for each strategy with the repeated measures ANOVA with one factor “Strategy.” The statistical analysis was performed for each graph theory measure. *Post-hoc* paired *t*-tests were performed to calculate the differences in the preprocessing steps. We corrected all the tests for multiple comparisons using a Bonferroni correction.

## Results

Overall, we found that **csr** (where we applied realignment, smoothing, and tCompCor as a final step) and **scr** (where we applied realignment, tCompCor, and smoothing as a final step) strategies had the highest mean values of ***eg***. However, we found that **fscr** strategy (where we applied realignment, tCompCor, smoothing, and filtering as a final step), had the highest mean values of ***el***.

### Effect of slice-timing on rs-fMRI

The graph theory measures ***el*** and ***eg*** had the same mean values in the **brut** and **a** strategies (Table 1). These results suggested that slice-timing, which is crucial for task-fMRI, may not be mandatory in the rs-fMRI studies at the TR that we used.

**Table 1:** Mean values of global and local efficiency for each strategy.

**Strategy**	***el***	***eg***	***Post-hoc t*-test for *el* # if *p* < 10^−3^**										
			*Post-hoc t*-test for eg^*^ if *p* < 10^−3^										
			**a**	**r**	**sr**	**cr**	**Scr**	**csr**	**fr**	**sfr**	**fcr**	**fscr**	**fcsr**
brut	0.592 ± 0.072	0.327 ± 0.068		^*^#	^*^#	^*^	^*^#	^*^#	^*^	^*^#	^*^#	^*^#	^*^#
a	0.587 ± 0.071	0.338 ± 0.079	–	^*^#	^*^#	^*^	^*^#	^*^#	^*^	^*^#	^*^#	^*^#	^*^#
r	0.645 ± 0.045	0.389 ± 0.051	–	–	=	^*^#	^*^	^*^			^*^	^*^#	^*^#
sr	0.660 ± 0.052	0.400 ± 0.052	–	–	–	^*^#	^*^	^*^	#		^*^	^*^#	^*^#
cr	0.582 ± 0.040	0.496 ± 0.020	–	–	–	–	^*^#	^*^#	^*^#	^*^#	#	^*^#	^*^#
scr	0.634 ± 0.027	**0.507 ± 0.014**	–	–	–	–	–	**#**	^*^	^*^	^*^	^*^#	^*^#
csr	0.644 ± 0.028	**0.503 ± 0.020**	–	–	–	–	–	–	^*^	^*^	^*^	^*^#	^*^#
fr	0.622 ± 0.048	0.397 ± 0.061	–	–	–	–	–	–	–	#	^*^	^*^#	^*^#
sfr	0.651 ± 0.051	0.407 ± 0.053	–	–	–	–	–	–	–	–	^*^	^*^#	^*^#
fcr	0.641 ± 0.029	0.485 ± 0.027	–	–	–	–	–	–	–	–	–	^*^#	^*^#
fscr	**0.691 ± 0.021**	0.466 ± 0.023	–	–	–	–	–	–	–	–	–	–	#
fcsr	0.681 ± 0.022	0.481 ± 0.019	–	–	–	–	–	–	–	–	–	–	–
**Repeated measure ANOVA**													
***p*-value**	*p* < 10^−5^	*p* < 10^−5^											
**F**	40.2	128.4											

*Values in BOLD indicates best strategies that provided significantly results than any other ones*.

### Effect of preprocessing steps on local and global efficiency for brain functional network

For each preprocessing strategy, ***el*** and ***eg*** were calculated. The curves of these measures for each network of each strategy were represented between the two curves of the random network and the regular network over the same range cost (Supplementary Tables 1, 2).

We found a significant effect of strategy for ***el*** (*p* < 10^−5^; *F* = 40.2) and for ***eg*** (*p* < 10^−5^, *F* = 128.4; Table 1). *Post-hoc t*-tests showed that for ***el***, there were no significant differences between (**cr=a=brut**), (**a=brut=fr**), (**r=sr=sfr=fcr=scr**), (**r=sr=sfr=fcr=csr**), (**r=fr=fcr=scr**) and (**r=fr=fcr=csr**). The mean values were ordered as follows: (**cr=a=brut**) < (**a=brut=fr**) < (**fr=scr=fcr=r**) < (**fr=fcr=csr=r**) < (**scr=fcr=r=sfr=sr**) < (**fcr=csr=r=sfr=sr**) < **fcsr** < **fscr**. Therefore, the highest mean values of ***el*** were observed in strategy **fscr** (el = 0.691 ± 0.021), and the *p*-values are presented in Supplementary Table 2.

*Post-hoc t-*tests showed that for ***eg***, each strategy was significantly different from the other strategies except for strategies (**brut=a**), (**r=fr=sr=sfr**), (**fcsr=fcr**) as well as (**fcr=cr**). The mean values were ordered as follows (**brut=a**) < (**r=fr=sr=sf**r) < **fscr** < (**fcsr=fcr**) < (**fcr=cr**) < (**csr=scr**). The highest mean values of ***eg*** were observed in the **csr** (e.g., = 0.503 ± 0.020) and **scr** (e.g., = 0.507 ± 0.014) strategies and the *p*-values are presented in Supplementary Table 2.

## Discussion

In this study, we evaluated the impact of the most common rs-fMRI preprocessing steps including slice-timing, realignment, filtering, smoothing, and tCompCor method on the graph theory measures by modifying the order and presence of each step in the preprocessing strategy in a group of healthy volunteers. We studied this impact on the topology of the graphs and their efficiencies (***eg*** and ***el***). We found that **csr** and **scr** strategies (where we applied realignment, smoothing and tCompCor) provided the highest values of ***eg***. Furthermore, we found that the **fscr** strategy (where we applied realignment, tCompCor, smoothing, and filtering as a final step) had the highest values of ***el***.

The majority of studies evaluated the effect of global signal regression on functional connectivity using graph theory and seed-based analysis. Fewer works evaluated the effects of other steps such as tComCor and filtering. We will discuss our results regarding these studies in the following paragraphs.

### Effect of slice-timing on local and global efficiency

We did not find any significant differences between the data with and without slice timing, for both ***el*** and ***eg***. A previous study evaluated the impact of slice-timing on functional connectivity estimated using correlation coefficients, the amplitude of low-frequency fluctuations (ALFFs) and fractional ALFF (fALFF) (Wu et al., 2011). They showed that slice-timing had no significant effect on correlation coefficients and fALFF using three different TRs (2, 3, and 4 s). However, ALFF increased significantly when slice-timing was applied at TR = 2 s. Although the metrics used in our study and the previous one differed, graph theory measures used here were derived from the correlation coefficients of the correlation matrix and therefore we may consider that both results agree.

### Effect of filtering on local and global efficiency

The comparison of the strategies with and without filtering (**r = fr**; **sr = sfr**; **cr = fcr**) showed no significant filtering effect for ***eg***. The highest ***eg*** values were observed for the **csr** and **scr** strategies. In contrast for ***el***, the highest values were observed for the **fcsr** and **fscr** strategies. These results suggest that filtering improved the local (***el)*** but not the global graph measures ***(eg)*** when the strategy included tCompCor. Overall, our results are in agreement with those of previous studies which suggested that filtering improved the local efficiency more than the global efficiency (Braun et al., 2012; Liang et al., 2012; Aurich et al., 2015; Borchardt et al., 2016). Previous studies have reported that the frequency of functional fluctuations of the spontaneous BOLD activity was in the range of 0.01–0.1 Hz (Biswal et al., 1995; Damoiseaux et al., 2006). This motivated our choice of frequency band. Previous rs-fMRI studies assessing the effect of filtering used a large range of frequency bands. Aurich et al. (2015) using the same frequency band (0.01–0.1 Hz) compared the graph theory measures for seven strategies and reported that strategies that included filtering provided higher local measures (Aurich et al., 2015). The application of the broad frequency band (0.008–0.15 Hz) also increased the reliability of the local measures (Braun et al., 2012). However, the broad frequency bands may pass frequencies corresponding to the physiological noise (Guijt et al., 2007). On the other hand, a broader frequency band may comprise more signals from the brain neural networks with different frequency bands (Buzsáki and Draguhn, 2004) than a narrower frequency band (0.027–0.073 Hz) which may eliminate signals of interest (Liang et al., 2012; Borchardt et al., 2016) Two studies suggested that the reliability of local measures was higher in the slow-4 band (0.027–0.073 Hz) than in the slow-5 band (0.01–0.027 Hz) (Liang et al., 2012; Borchardt et al., 2016). Specific investigation of the optimal frequency band on graph theory measures needs to be further investigated.

### Effect of smoothing on local and global efficiency

Smoothing also increased ***el***. The highest values of ***el*** were observed in strategies (**sfr**, **sr**, **fcsr**, and **fscr**). The smoothing was a Gaussian spatial filter, which is a standard preprocessing step used in most studies. Previous studies have shown that smoothing increased the reproducibility of the local efficiency but had no effect on global efficiency (Telesford et al., 2010). Smoothing also increased the functional connectivity estimated using three indices, i.e., correlation coefficients, (ALFFs) and fractional ALFF (fALFF), when using seed-based analysis (Wu et al., 2011). These two studies agree because an increase in the functional connectivity indicated an increase in the correlation coefficients, which probably resulted in increased clustering and local efficiency (***el***). Our findings in the strategies including smoothing (**sfr**, **sr**, **fcsr**, and **fscr**) are in line with these results. Lastly, smoothing also increased global efficiency (***eg***) when associated with tCompCor as shown by the (**scr** and **csr**) strategies.

### Effect of tcompcor on local and global efficiency

tCompCor estimates the noise in the BOLD signal time-course using tSTD of the voxels with the highest tSTD values (Behzadi et al., 2007). In our study, we found that strategies (**cr**, **scr**, and **csr**), in which tComCor was applied with or without smoothing, increased ***eg***. In contrast, the lowest ***el*** values were observed when tCompCor was applied without smoothing or filtering (strategy **cr**). The ***el*** value increased only when tCompCor was applied with filtering or smoothing or both of them. To our knowledge, no study has evaluated the effect of tCompCor on graph theory measures. However, the impact of global signal regression has been extensively studied (Murphy et al., 2009; Braun et al., 2012; Guo et al., 2012; Liang et al., 2012; Jo et al., 2013). As detailed in the introduction, these studies found that global signal regression did not improve functional connectivity and graph theory measures (Murphy et al., 2009; Braun et al., 2012; Guo et al., 2012; Liang et al., 2012; Jo et al., 2013). In sum, strategies including tCompCor provided better ***eg*** values, whereas higher ***el*** values were obtained when adding filtering or smoothing. Lastly, tCompCor was easy to apply and did not require external monitoring of physiological fluctuations.

## Limitations

Identifying of the best strategy for the preprocessing of rs-fMRI data is difficult. This choice depends on the number of subjects, the number of regions constructing the networks and the final objective of the study. In our project we evaluated 12 strategies including smoothing, tCompCor, and filtering with a different order of application and proposed an optimal order of processing steps. Our result may not apply to other data analysis method. Further study is thus needed to extend these results to other datasets with different acquisition parameters and data analysis methods. Controlling the quality of rs-fMRI is a crucial step but remains insufficiently studied. In addition, a similar impact may be expected in pathological subjects.

## Conclusion

Tests that we conducted in healthy subjects showed that the choice and ordering of the preprocessing steps impacted the graph theory measures. Overall, our results confirmed that graph theory measures of functional connectivity depend on the ordering of the processing steps. They also suggested that global efficiency was improved when smoothing and tCompCor were applied as the final steps of the preprocessing pipeline and that local efficiency was improved by additional filtering.

## Ethics statement

This study was carried out in accordance with the recommendations of Institut du cerveau et de la moelle & pini re (ICM) with written informed consent from all subjects. All subjects gave written informed consent in accordance with the Declaration of Helsinki. The protocol was approved by the ICM.

## Author contributions

FG: make substantial contributions to conception, design, analysis, and interpretation of data; FK: participate in analysis and interpretation of data; SD: participate in analysis and interpretation of data; AB: participate in revising the article; SL: participate in drafting the article and revising it for important intellectual content and give the final approval of the version to be submitted; RV: participate in analysis and interpretation of data.

### Conflict of interest statement

The authors declare that the research was conducted in the absence of any commercial or financial relationships that could be construed as a potential conflict of interest.

## Supplementary material

Supplementary Table 1 The threshold, the small-worldness, the local efficiency and the global efficiency per density for each strategy.

Supplementary Table 2 The *p*-values for the *t*-tests between all strategies.
