# Supplementary material for: The Influence of Preprocessing Steps on Graph Theory Measures Derived from Resting State fMRI
Source: Front Comput Neurosci. 2018 Feb 13;12:8. doi: 10.3389/fncom.2018.00008 (PMC5819575; doi:10.3389/fncom.2018.00008)
Supplement: Supplementary Table 1 — The threshold, the small-worldness, the local efficiency and the global efficiency per density for each strategy. [file Table1.pdf]

Threshold per density for each strategy

density/cost		0.0413	0.0513	0.0614	0.0714	0.0814	0.0914	0.1015	0.1115	0.1215	0.1315	0.1416	0.1516	0.1616	0.1717	0.1817	0.1917	0.2017	0.2118	0.2218	0.2318	0.2418	0.2519
threshold	brut	0.7909	0.7753	0.7614	0.7485	0.7367	0.7257	0.7153	0.7055	0.6956	0.6864	0.6775	0.6687	0.6603	0.6521	0.6441	0.6363	0.6287	0.6211	0.6138	0.6064	0.5993	0.5922
	a	0.7920	0.7763	0.7624	0.7496	0.7378	0.7267	0.7162	0.7062	0.6966	0.6874	0.6786	0.6701	0.6617	0.6538	0.6457	0.6379	0.6301	0.6226	0.6152	0.6080	0.6008	0.5937
	r	0.6666	0.6505	0.6365	0.6238	0.6125	0.6021	0.5924	0.5833	0.5747	0.5665	0.5586	0.5509	0.5436	0.5362	0.5293	0.5226	0.5160	0.5096	0.5033	0.4971	0.4910	0.4851
	sr	0.8065	0.7956	0.7858	0.7771	0.7690	0.7615	0.7546	0.7480	0.7416	0.7356	0.7298	0.7241	0.7184	0.7129	0.7077	0.7025	0.6975	0.6925	0.6877	0.6829	0.6782	0.6734
	cr	0.2981	0.2819	0.2686	0.2571	0.2471	0.2381	0.2300	0.2226	0.2156	0.2093	0.2036	0.1980	0.1926	0.1876	0.1829	0.1784	0.1740	0.1699	0.1658	0.1620	0.1582	0.1546
	csr	0.3987	0.3781	0.3613	0.3466	0.3337	0.3222	0.3118	0.3021	0.2932	0.2849	0.2772	0.2699	0.2630	0.2565	0.2503	0.2442	0.2384	0.2329	0.2276	0.2226	0.2176	0.2128
	scr	0.4244	0.4026	0.3847	0.3693	0.3555	0.3432	0.3319	0.3218	0.3123	0.3035	0.2951	0.2872	0.2798	0.2729	0.2663	0.2600	0.2539	0.2480	0.2424	0.2370	0.2317	0.2267
	fr	0.7542	0.7401	0.7281	0.7167	0.7066	0.6971	0.6882	0.6794	0.6712	0.6636	0.6560	0.6490	0.6418	0.6351	0.6284	0.6217	0.6151	0.6088	0.6026	0.5965	0.5904	0.5843
	sfr	0.8535	0.8436	0.8348	0.8268	0.8193	0.8122	0.8056	0.7991	0.7932	0.7873	0.7815	0.7758	0.7704	0.7650	0.7599	0.7548	0.7499	0.7450	0.7403	0.7355	0.7307	0.7261
	fcr	0.4610	0.4357	0.4144	0.3954	0.3777	0.3618	0.3474	0.3334	0.3204	0.3082	0.2968	0.2858	0.2755	0.2654	0.2558	0.2464	0.2374	0.2286	0.2202	0.2115	0.2033	0.1953
	fcsr	0.5255	0.4970	0.4732	0.4517	0.4321	0.4143	0.3978	0.3825	0.3682	0.3544	0.3415	0.3289	0.3171	0.3059	0.2950	0.2843	0.2740	0.2640	0.2543	0.2448	0.2353	0.2263
	fscr	0.5618	0.5326	0.5077	0.4855	0.4654	0.4457	0.4283	0.4118	0.3964	0.3818	0.3676	0.3540	0.3410	0.3286	0.3166	0.3051	0.2940	0.2829	0.2721	0.2617	0.2516	0.2417

Small-woldness per density for each strategy

small-worldness																						
brut	8.8030	7.2536	6.2426	5.4743	4.9234	4.4814	4.1339	3.8452	3.6092	3.3977	3.2418	3.1046	2.9871	2.8876	2.7924	2.7074	2.6077	2.5313	2.4755	2.4191	2.3613	2.3037
a	8.8025	7.3281	6.3163	5.5321	4.9540	4.4877	4.1265	3.8620	3.6128	3.4169	3.2301	3.0921	2.9963	2.8868	2.7743	2.6881	2.6233	2.5545	2.4805	2.4438	2.3745	2.3175
r	8.4429	7.1234	6.2415	5.6123	5.1019	4.6395	4.2524	3.9287	3.6660	3.4915	3.3197	3.1798	3.0520	2.9455	2.8504	2.7664	2.6814	2.5998	2.5337	2.4657	2.4003	2.3472
sr	7.7287	6.5310	5.6974	5.1258	4.6131	4.2447	3.9152	3.6663	3.4329	3.2526	3.1036	2.9823	2.8492	2.7570	2.6652	2.6098	2.5261	2.4613	2.3991	2.3449	2.2972	2.2460
cr	6.8487	5.6938	4.9183	4.3552	3.8661	3.4857	3.1718	2.9144	2.7094	2.5366	2.3970	2.2821	2.1799	2.0879	2.0042	1.9280	1.8660	1.8042	1.7442	1.6932	1.6436	1.6009
csr	7.3573	6.2306	5.4241	4.8252	4.3384	3.9038	3.5384	3.2482	3.0166	2.8314	2.6720	2.5383	2.4178	2.3111	2.2109	2.1249	2.0419	1.9718	1.9027	1.8408	1.7808	1.7253
scr	7.4818	6.3615	5.5417	4.9428	4.4441	4.0099	3.6417	3.3822	3.1335	2.9463	2.7744	2.6392	2.5124	2.4050	2.3132	2.2287	2.1436	2.0685	1.9967	1.9313	1.8674	1.8122
fr	7.7108	6.4155	5.5602	4.9364	4.4980	4.0880	3.7947	3.5457	3.3264	3.1408	2.9949	2.8794	2.7690	2.6603	2.5701	2.5030	2.4380	2.3836	2.3331	2.2767	2.2141	2.1659
sfr	7.4075	6.0839	5.4122	4.8816	4.4191	4.0549	3.7540	3.5163	3.3191	3.1362	2.9701	2.8616	2.7445	2.6668	2.5978	2.5359	2.4756	2.4107	2.3538	2.2862	2.2331	2.1901
fcr	6.1270	5.2284	4.6798	4.2363	3.8824	3.6147	3.3397	3.0925	2.9175	2.7586	2.6201	2.5099	2.4165	2.3320	2.2616	2.1911	2.1274	2.0689	2.0159	1.9667	1.9215	1.8774
fcsr	6.4975	5.6849	5.1596	4.6769	4.2826	3.9137	3.6218	3.3669	3.1463	2.9767	2.8189	2.6908	2.5853	2.4846	2.4004	2.3218	2.2499	2.1856	2.1302	2.0733	2.0222	1.9746
fscr	6.5940	5.6198	5.0521	4.5516	4.1753	3.8698	3.6076	3.3860	3.1904	3.0286	2.8921	2.7615	2.6494	2.5538	2.4705	2.4007	2.3285	2.2615	2.2005	2.1409	2.0863	2.0356
density	0.0413	0.0513	0.0614	0.0714	0.0814	0.0914	0.1015	0.1115	0.1215	0.1315	0.1416	0.1516	0.1616	0.1717	0.1817	0.1917	0.2017	0.2118	0.2218	0.2318	0.2418	0.2519

Local efficiency per density for each strategy

local efficiency																						
random	0.0453	0.0614	0.0789	0.1086	0.1380	0.1819	0.2309	0.2816	0.3350	0.3817	0.4226	0.4570	0.4846	0.5083	0.5293	0.5464	0.5611	0.5740	0.5863	0.5973	0.6066	0.6155
régulier	0.6453	0.7465	0.7909	0.7956	0.8151	0.8229	0.8282	0.8363	0.8367	0.8444	0.8458	0.8498	0.8524	0.8540	0.8570	0.8573	0.8605	0.8612	0.8633	0.8645	0.8656	0.8671
brut	0.3567	0.3932	0.4276	0.4573	0.4854	0.5082	0.5312	0.5520	0.5725	0.5894	0.6071	0.6231	0.6374	0.6523	0.6653	0.6764	0.6863	0.6965	0.7086	0.7190	0.7277	0.7362
a	0.3538	0.3931	0.4261	0.4569	0.4827	0.5044	0.5266	0.5482	0.5674	0.5864	0.6018	0.6162	0.6331	0.6460	0.6581	0.6708	0.6838	0.6950	0.7051	0.7167	0.7242	0.7324
r	0.4081	0.4558	0.4912	0.5242	0.5537	0.5780	0.5983	0.6175	0.6357	0.6513	0.6647	0.6788	0.6920	0.7032	0.7133	0.7244	0.7311	0.7379	0.7474	0.7558	0.7610	0.7684
sr	0.4297	0.4743	0.5117	0.5443	0.5737	0.5986	0.6193	0.6389	0.6541	0.6682	0.6811	0.6943	0.7042	0.7144	0.7237	0.7351	0.7438	0.7515	0.7585	0.7670	0.7743	0.7787
cr	0.3797	0.4206	0.4541	0.4827	0.5052	0.5271	0.5457	0.5614	0.5766	0.5894	0.6018	0.6131	0.6241	0.6339	0.6426	0.6503	0.6581	0.6649	0.6710	0.6779	0.6830	0.6887
csr	0.4560	0.4994	0.5336	0.5607	0.5848	0.6017	0.6152	0.6261	0.6379	0.6478	0.6564	0.6649	0.6718	0.6770	0.6821	0.6874	0.6916	0.6957	0.6995	0.7034	0.7066	0.7094
scr	0.4645	0.5094	0.5431	0.5697	0.5942	0.6102	0.6221	0.6369	0.6469	0.6567	0.6644	0.6725	0.6792	0.6851	0.6918	0.6979	0.7018	0.7059	0.7095	0.7133	0.7164	0.7199
fr	0.3821	0.4284	0.4648	0.4956	0.5234	0.5472	0.5721	0.5913	0.6095	0.6244	0.6396	0.6544	0.6705	0.6802	0.6930	0.7036	0.7126	0.7225	0.7323	0.7412	0.7468	0.7539
sfr	0.4052	0.4535	0.4959	0.5296	0.5592	0.5821	0.6025	0.6227	0.6404	0.6559	0.6695	0.6849	0.6976	0.7104	0.7227	0.7329	0.7430	0.7501	0.7582	0.7631	0.7697	0.7766
fcr	0.4092	0.4570	0.4970	0.5321	0.5625	0.5912	0.6109	0.6273	0.6436	0.6576	0.6683	0.6793	0.6886	0.6967	0.7051	0.7112	0.7167	0.7223	0.7270	0.7316	0.7362	0.7403
fcsr	0.4688	0.5205	0.5654	0.6009	0.6272	0.6471	0.6634	0.6762	0.6870	0.6989	0.7066	0.7139	0.7216	0.7264	0.7320	0.7369	0.7412	0.7452	0.7496	0.7525	0.7560	0.7591
fscr	0.4784	0.5306	0.5751	0.6043	0.6295	0.6495	0.6685	0.6855	0.6979	0.7104	0.7204	0.7268	0.7328	0.7384	0.7445	0.7507	0.7549	0.7589	0.7625	0.7652	0.7683	0.7711
density	0.0413	0.0513	0.0614	0.0714	0.0814	0.0914	0.1015	0.1115	0.1215	0.1315	0.1416	0.1516	0.1616	0.1717	0.1817	0.1917	0.2017	0.2118	0.2218	0.2318	0.2418	0.2519

Global efficiency per density for each strategy

global efficiency																						
random	0.3835	0.4178	0.4447	0.4679	0.4886	0.5069	0.5228	0.5363	0.5477	0.5573	0.5654	0.5726	0.5789	0.5848	0.5902	0.5955	0.6007	0.6058	0.6109	0.6159	0.6209	0.6259
régulier	0.0867	0.1024	0.1172	0.1313	0.1447	0.1575	0.1699	0.1818	0.1934	0.2046	0.2154	0.2260	0.2363	0.2463	0.2561	0.2657	0.2750	0.2842	0.2931	0.3019	0.3105	0.3189
brut	0.1263	0.1517	0.1753	0.1979	0.2206	0.2424	0.2623	0.2813	0.3008	0.3192	0.3368	0.3538	0.3702	0.3857	0.4013	0.4158	0.4293	0.4442	0.4571	0.4703	0.4833	0.4966
a	0.1263	0.1519	0.1749	0.1973	0.2197	0.2407	0.2606	0.2801	0.2981	0.3166	0.3342	0.3505	0.3664	0.3824	0.3976	0.4135	0.4279	0.4415	0.4554	0.4676	0.4807	0.4924
r	0.1697	0.2015	0.2306	0.2574	0.2829	0.3076	0.3287	0.3489	0.3690	0.3860	0.4023	0.4191	0.4354	0.4501	0.4636	0.4770	0.4893	0.5012	0.5132	0.5253	0.5363	0.5469
sr	0.1880	0.2223	0.2518	0.2774	0.3025	0.3248	0.3458	0.3648	0.3830	0.3998	0.4152	0.4293	0.4435	0.4568	0.4698	0.4822	0.4943	0.5068	0.5177	0.5277	0.5377	0.5479
cr	0.2637	0.3098	0.3522	0.3846	0.4143	0.4408	0.4633	0.4828	0.5006	0.5156	0.5298	0.5413	0.5526	0.5627	0.5722	0.5809	0.5889	0.5963	0.6035	0.6103	0.6167	0.6226
csr	0.2831	0.3311	0.3685	0.3987	0.4260	0.4488	0.4682	0.4852	0.5013	0.5159	0.5288	0.5409	0.5523	0.5623	0.5718	0.5803	0.5884	0.5960	0.6029	0.6095	0.6159	0.6220
scr	0.2624	0.3069	0.3477	0.3787	0.4074	0.4317	0.4529	0.4715	0.4892	0.5044	0.5177	0.5309	0.5433	0.5543	0.5644	0.5740	0.5827	0.5909	0.5989	0.6062	0.6131	0.6196
fr	0.1745	0.2089	0.2394	0.2668	0.2906	0.3140	0.3351	0.3542	0.3742	0.3912	0.4082	0.4234	0.4391	0.4535	0.4679	0.4809	0.4931	0.5043	0.5157	0.5266	0.5372	0.5477
sfr	0.1789	0.2161	0.2481	0.2758	0.3013	0.3244	0.3458	0.3656	0.3842	0.4025	0.4186	0.4339	0.4508	0.4650	0.4779	0.4901	0.5022	0.5142	0.5254	0.5361	0.5468	0.5562
fcr	0.2443	0.2931	0.3332	0.3671	0.3946	0.4187	0.4405	0.4596	0.4765	0.4913	0.5054	0.5183	0.5302	0.5418	0.5523	0.5624	0.5717	0.5808	0.5892	0.5974	0.6050	0.6123
fcsr	0.2470	0.2947	0.3327	0.3651	0.3920	0.4154	0.4359	0.4540	0.4705	0.4855	0.4996	0.5125	0.5243	0.5355	0.5460	0.5560	0.5655	0.5746	0.5833	0.5918	0.5997	0.6074
fscr	0.2159	0.2650	0.3063	0.3395	0.3694	0.3948	0.4163	0.4364	0.4551	0.4712	0.4861	0.5001	0.5131	0.5255	0.5369	0.5476	0.5575	0.5673	0.5765	0.5853	0.5938	0.6021
density	0.0413	0.0513	0.0614	0.0714	0.0814	0.0914	0.1015	0.1115	0.1215	0.1315	0.1416	0.1516	0.1616	0.1717	0.1817	0.1917	0.2017	0.2118	0.2218	0.2318	0.2418	0.2519
