# Supplementary material for: The Influence of Preprocessing Steps on Graph Theory Measures Derived from Resting State fMRI
Source: Front Comput Neurosci. 2018 Feb 13;12:8. doi: 10.3389/fncom.2018.00008 (PMC5819575; doi:10.3389/fncom.2018.00008)
Supplement: Supplementary Table 2 — The p-values for the t-tests between all strategies. [file Table2.pdf]

P-values

el

effect	DFn	DFd	SSn	SSd	F	p
strategie	1	49	0.3397	0.241	69.07	6.40E-11

ttest

	brut	a	r	sr	cr	scr	csr	fr	sfr	fcr	fscr
a	1	NA	NA	NA	NA	NA	NA	NA	NA	NA	NA
r	0.00035	8.40E-06	NA	NA	NA	NA	NA	NA	NA	NA	NA
sr	7.50E-06	3.20E-07	0.2	NA	NA	NA	NA	NA	NA	NA	NA
cr	1	1	1.40E-10	2.10E-11	NA	NA	NA	NA	NA	NA	NA
scr	0.0029	0.001	1	0.07	1.30E-17	NA	NA	NA	NA	NA	NA
csr	7.50E-05	3.60E-05	1	1	4.90E-25	7.00E-04	NA	NA	NA	NA	NA
fr	1	3.90E-01	0.94	0.005	0.001	1	0.43	NA	NA	NA	NA
sfr	0.001	2.00E-04	1	1	1.01E-07	1	1	2.61E-09	NA	NA	NA
fcr	0.0009	2.00E-04	1	0.9	2.80E-15	1	1	0.44	1	NA	NA
fscr	5.63E-12	1.46E-12	7.55E-07	4.00E-03	1.71E-25	7.07E-21	1.77E-18	3.69E-12	9.49E-06	8.74E-23	NA
fcsr	2.95E-10	1.14E-10	7.83E-05	2.50E-01	9.46E-25	7.56E-20	6.01E-14	1.89E-10	3.00E-03	2.34E-14	4.00E-03

eg

effect	DFn	DFd	SSn	SSd	F	p
strategie	1	49	1.02	0.288	173.4	1.02E-17

ttest

	brut	a	r	sr	cr	scr	csr	fr	sfr	fcr	fscr
a	0.08	NA	NA	NA	NA	NA	NA	NA	NA	NA	NA
r	3.24E-05	4.76E-06	NA	NA	NA	NA	NA	NA	NA	NA	NA
sr	4.37E-07	2.72E-08	1	NA	NA	NA	NA	NA	NA	NA	NA
cr	4.14E-20	2.13E-20	1.03E-15	8.03E-15	NA	NA	NA	NA	NA	NA	NA
scr	1.38E-20	8.66E-21	2.87E-18	6.13E-17	5.00E-03	NA	NA	NA	NA	NA	NA
csr	2.68E-20	9.07E-21	2.66E-16	1.14E-15	1.00E-02	1.00E+00	NA	NA	NA	NA	NA
fr	3.00E-04	4.69E-05	1.00E+00	1.00E+00	6.64E-13	4.27E-15	1.92E-15	NA	NA	NA	NA
sfr	5.28E-06	4.46E-07	1.00E+00	1.00E+00	1.10E-13	6.68E-16	1.49E-16	4.80E-01	NA	NA	NA
fcr	1.55E-17	5.79E-18	1.26E-13	1.09E-12	6.00E-02	2.91E-05	4.66E-07	1.14E-11	5.40E-12	NA	NA
fscr	5.72E-16	2.11E-16	1.05E-10	3.66E-09	5.71E-17	2.73E-15	1.14E-18	6.10E-08	8.91E-08	1.53E-14	NA
fcsr	5.12E-18	1.67E-18	2.58E-14	1.50E-12	1.00E-02	6.36E-14	2.85E-06	4.26E-11	3.05E-11	1.00E+00	3.00E-03
